# Periodontal disease and cancer risk: A nationwide population-based cohort study

**DOI:** 10.3389/fonc.2022.901098

**Published:** 2022-08-23

**Authors:** Eun Hwa Kim, Sunghyun Nam, Chung Hyun Park, Yitak Kim, Myeongjee Lee, Joong Bae Ahn, Sang Joon Shin, Yu Rang Park, Hoi In Jung, Baek-Il Kim, Inkyung Jung, Han Sang Kim

**Affiliations:** ^1^ Biostatistics Collaboration Unit, Department of Biomedical Systems Informatics, Yonsei University College of Medicine, Seoul, South Korea; ^2^ College of Medicine, Yonsei University, Seoul, South Korea; ^3^ Department of Internal Medicine, Yonsei University College of Medicine, Seoul, South Korea; ^4^ Yonsei Cancer Center, Division of Medical Oncology, Department of Internal Medicine, Yonsei University College of Medicine, Seoul, South Korea; ^5^ Department of Preventive Dentistry and Public Oral Health, BK21 FOUR Project, Yonsei University College of Dentistry, Seoul, South Korea; ^6^ Division of Biostatistics, Department of Biomedical Systems Informatics, Yonsei University College of Medicine, Seoul, South Korea; ^7^ Graduate School of Medical Science, Brain Korea 21 Project, Severance Biomedical Science Institute, Yonsei University College of Medicine, Seoul, South Korea; ^8^ Institute for Innovation in Digital Healthcare (IIDH), Severance Hospital, Seoul, South Korea

**Keywords:** periodontal disease, cancer risk, cohort study, periodontitis, oral inflammation

## Abstract

**Background:**

Although emerging evidence suggests that periodontitis might increase the risk of cancer, comorbidity and lifestyle behaviors, such as smoking and body mass index (BMI), may have confounded this reported association. This study aimed to investigate whether chronic periodontitis is associated with cancer risk using a large, nationwide database.

**Methods:**

We conducted a population-based, retrospective cohort study using data from the Korean National Health Insurance Cohort Database obtained between January 2003 and December 2015. We included 713,201 individuals without a history of cancer who were followed up to 10 years. Confounding factors included demographic factors (age, sex, income, and residential area), lifestyle behaviors (smoking history and BMI), and comorbidities, such as hypertension, diabetes, heart failure, and pulmonary disease, using the Charlson Comorbidity Index. Multivariable Cox regression analysis was applied to estimate the adjusted hazard ratio (aHR) for cancer risk.

**Results:**

Of the 713,201 participants, 53,075 had periodontitis and were placed in the periodontitis group; the remaining 660,126 individuals were included as the control group. Overall, the cumulative incidence of cancer in the periodontitis group was 2.2 times higher than that in the control group. The periodontitis group had an increased risk of total cancer compared to the control group after adjusting for age, sex, comorbidities, BMI, and smoking history (aHR, 1.129; 95% confidence interval [CI], 1.089-1.171; *P*<0.0001). When examining specific cancer types, significant associations were also observed between periodontitis and stomach cancer (aHR, 1.136; 95% CI, 1.042-1.239; *P*=0.0037), colon cancer (aHR, 1.129; 95% CI, 1.029-1.239; *P*=0.0105), lung cancer (aHR, 1.127; 95% CI, 1.008-1.260; *P*=0.0353), bladder cancer (aHR, 1.307; 95% CI, 1.071-1.595; *P=*0.0085), thyroid cancer (aHR, 1.191; 95% CI, 1.085-1.308; *P=*0.0002), and leukemia (aHR, 1.394; 95% CI, 1.039-1.872; *P*=0.0270). There was no significant association between the development of secondary malignancy and periodontitis in cancer survivors who were alive 5 years after they were diagnosed with the primary malignancy.

**Conclusions:**

Periodontal disease, including periodontitis, was associated with increased risk of cancer, which persisted after controlling for confounding factors. Further prospective research is warranted to establish a causal relationship.

## Introduction

Periodontal disease is an inflammatory disorder of the periodontal tissue induced by dysbiotic plaque. It can range from a mild form, such as gingivitis, to a more severe, destructive form, such as periodontitis, which occurs as a result of the destruction of the attachment apparatus, including the alveolar bone, the periodontal ligament that subsequently to tooth loss  (1, 2). Periodontitis is an evolving disease and a recently updated classification framework based on a staging and grading system incorporating severity, tooth loss, and management complexity  (3). The global prevalence of periodontal disease is 20-50%, and approximately 10% of the global population is affected by severe periodontitis  (4–6). Recently, intensive efforts have been made to elucidate the effects of the dysbiotic oral microbiome on various systemic diseases, including cardiovascular disease and cancer   (7).

Previous observational reports and meta-analyses reported that the presence of periodontal disease positively correlates with an increased risk of total cancer and site-specific cancers  (8–12). Recent prospective studies have reported increases in the overall cancer risk associated with periodontal disease of 14% to 24%, and the association was not attenuated even after adjustment for known risk factors, such as smoking  (10, 12, 13). Although the methodology to define periodontal disease is not consistent across studies, multiple population-based studies have shown a consistent relationship between periodontitis and cancer risk, and the risk seems to increase significantly in proportion to disease severity (13). However, our understanding of the relationship between periodontal disease and site-specific cancer risk is limited, which makes it difficult to reach a consensus. A meta-analysis that reviewed 14 cohort and 20 case-control studies reported positive associations between periodontitis and oral, lung, and pancreatic cancers  (12). Other recent cohort studies have shown positive associations between periodontitis and esophageal, breast, lung, gallbladder, and colorectal cancers and melanoma  (10, 13). However, these conflicting relationships in specific tumor types may also be explained by the differences in study populations, cohort sizes, study designs, particularly the use of various clinical measures to classify periodontal disease, and the statistical effects of confounding variables.

Few prospective studies have investigated the relationship between periodontitis and overall and site-specific cancer incidences. In the present study, we aimed to examine the association between periodontal disease, including periodontitis, and the risks of total and site-specific cancers using the National Health Insurance Service-Health Examine Cohort data. Furthermore, we evaluated whether the risk of developing a secondary cancer would be different in patients with periodontal disease, including periodontitis.

## Materials and methods

### Study participants and design

**Figure 1:**
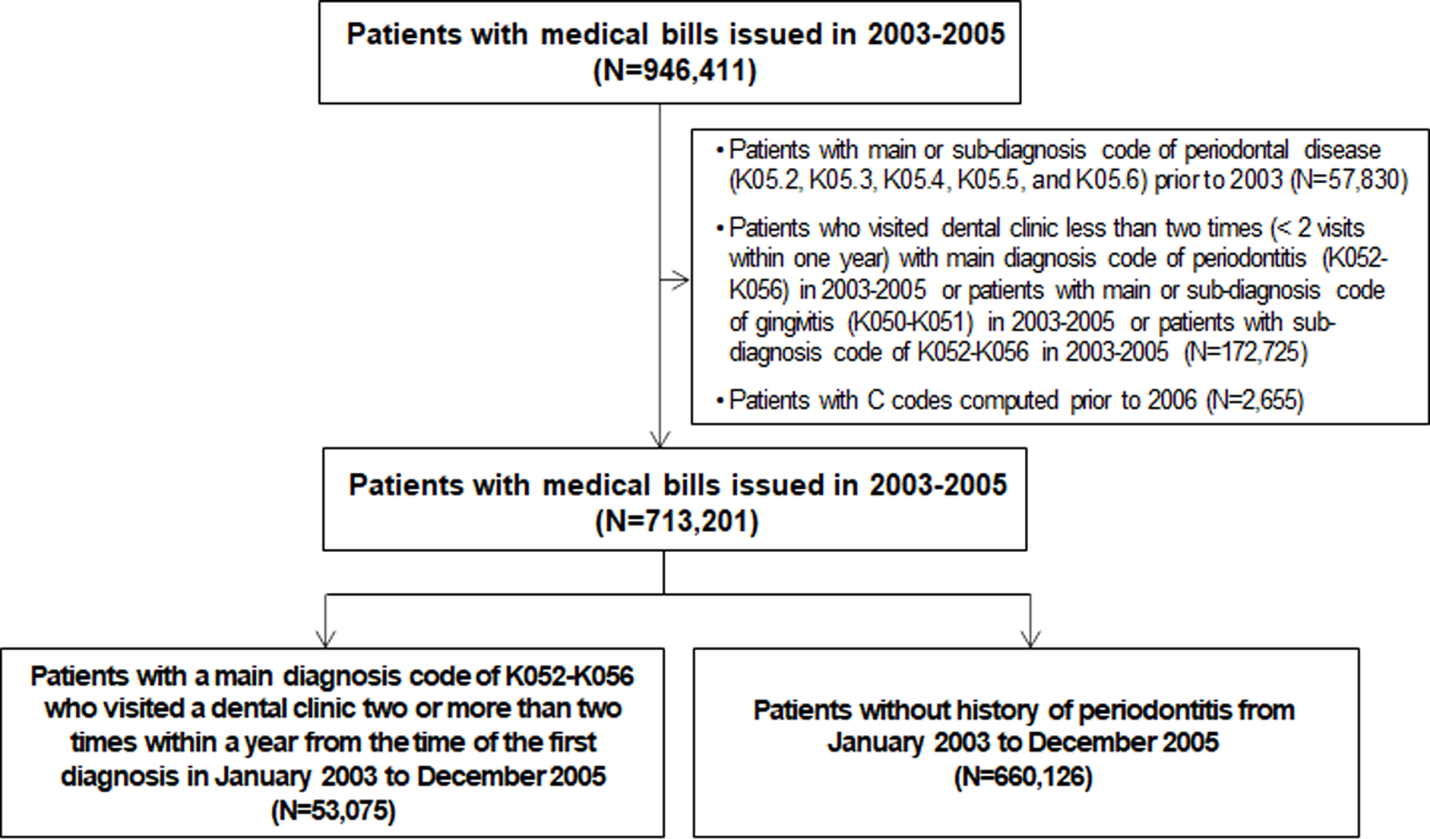
Flowchart of selection of the study population from the National Health Insurance Service-Health Screening Cohort database (n=713,201).

We conducted a population-based, retrospective cohort study using data from the Korean National Health Insurance Cohort Database obtained between January 2003 and December 2015. All patients in the database older than 1 year of age were included in the cohort. Patients diagnosed with any form of cancer during the washout period (2003-2005) were excluded. Those without a cancer history who visited a dental clinic two or more than two times within one year and were diagnosed with periodontitis under those ICD-10 codes (K05.2, K05.3, K05.4, K05.5, and K05.6) between January 2003 and December 2005, were included in the periodontitis group. As a control group, subjects have no history of periodontitis between 2003 and 2015. We excluded patients receiving a periodontal diagnosis prior to 2003 in the cohorts (**Figure 1**). A dentist performed an oral examination, and periodontitis was assessed using the Community Periodontal Index (CPI). Periodontal disease was defined as a CPI score≥3. The study population was followed up from the index date (January 2006) to the date of cancer, death, or the end of the study (December 2015). The study was approved by the Institutional Review Board (4-2019-0616).

### Study outcomes and definitions

The main outcome of this study was the association between periodontitis and overall and site-specific cancer risks. As certain types of cancers, such as breast cancer and cancers of reproductive organs, were not differentiated in the Korean National Health Insurance Database, these were placed under the category of “others.” These included breast, cervical, vulvar, vaginal, ovarian, and endometrial cancers in female patients and prostate, testicular, and penile cancers in male patients. The International Statistical Classification of Diseases and Related Health Problems (ICD-10) codes were used to designate the patients diagnosed with periodontal disease (K05.2 through K05.6) and those diagnosed with cancer, defined as a new claim for primary diagnosis of cancer (C code and V193). Patients with newly diagnosed cancer were registered with a special certification, code V193, from September 2005 for expanded benefit coverage in Korea.

The cancer occurrence date was defined as the date of a newly developed primary cancer from the National Health Insurance System (NHIS) database. The presence of periodontal disease was identified when ICD-10 codes for acute periodontitis (K05.2), chronic periodontitis (K05.3), periodontitis (K05.4), other periodontal diseases (K05.5), or unspecified periodontal disease (K05.6), as previous studies   (14, 15). Other periodontal diseases (K05.5) or unspecified periodontal diseases (K05.6) can include acute or chronic periodontitis (K05.2, K05.3, and K05.4).

To evaluate the development of secondary cancer, we evaluated whether the cancer survivors were diagnosed with another primary cancer different from the primary cancer type based on ICD-10 codes using C codes representing cancer diagnosis. Individuals who had not survived five years from their first primary cancer were excluded.

### Confounding variables

Confounding factors included demographic factors (age, sex, income, and residential area), lifestyle behaviors (smoking history and body mass index [BMI]), and comorbidities, as defined by the Charlson Comorbidity Index (CCI) (16). A healthy examination questionnaire obtained smoking status in the patient cohort between 2003 and 2005. Smoking status was categorized into none, former smoker, and current smoker regardless of the amount of smoking. CCI was calculated based on the ICD-10 codes according to previous studies  (17, 18). Briefly, CCI corresponds to the sum of the weights of the current comorbidities for each patient. Comorbidities with corresponding weights include myocardial infarction within the six months prior to surgery (1), congestive heart failure (1), peripheral vascular disease or rest pain (1), any history of cerebrovascular accident (1), dementia (1), chronic obstructive pulmonary disease (1), connective tissue disease (1), peptic ulcer disease (1), diabetes mellitus (1), moderate to severe chronic kidney disease (2), hemiplegia (2), leukemia (2), malignant lymphoma (2), ascites or esophageal varices (3), disseminated cancer (6), and acquired immune deficiency syndrome (6). The point values were summed for a total number.

### Statistical analysis

The Kolmogorov-Smirnov normality test was used to evaluate if variables are normally distributed. Continuous variables were expressed as median with interquartile range (IQR) and compared using the Mann–Whitney U test when the data did not follow the normal distribution. Categorical variables, such as sex, level of income, or residential area, were presented as numbers (%) and were compared using the chi‐squared test or Fisher’s exact test. Regarding categorical variables with missing values (such as BMI and smoking status), we treated missing values as a valid missing category. The Kaplan-Meier method was used to estimate the cumulative risk of cancer. Multivariable Cox proportional hazards regression analysis was used to estimate the adjusted hazard ratio (aHR) and 95% confidence intervals (CIs). The model was adjusted for potential confounders such as age, sex, comorbidities, BMI, and smoking history. Since the 77% of patients had missing value for BMI and smoking history, missing value were grouped into a “missing” category. Analyses were performed using the SAS Enterprise Guide version 7.1 (SAS Institute, Inc., Cary, NC, USA) and two-sided *P* value < 0.05 was considered statistically significant.

## Results

### Baseline characteristics of the study population

Among the 713,201 participants, 53,075 (7.4%) had periodontitis, and 660,126 (92.6%) were included as healthy controls between January 2003 to December 2005 (**Figure 1**). In the subjects with periodontitis (n=53,075), the median age was 49 years; 49.6% were males; 6.4% had BMI more than 25; 10.8% were current smokers (**Table 1).** Current smokers were nearly twice in the periodontitis group compared to the control (10.8% vs. 5.5%, respectively). Regarding socioeconomic status, the proportions of people living below or equal to 50% of the median income and people living at 51-80% of the median income were 3.7% and 2.2% higher in the control group than in the periodontitis group, respectively. In contrast, the proportion of people living above or equal to 80% of the median income was 6.0% higher in the periodontitis group (25.7%) than in the control group (31.7%). Both groups had nearly half of the participants living in the capital or metropolitan cities (control: 46.1% and periodontitis: 48.9%), respectively. Multivariate Cox regression analyses of potential confounding factors for cancer development in this study cohort (n=713,201) was shown in **Supplementary Table 1**. Female sex (aHR, 0.761; 95% CI, 0.740-0.783; *P* < 0.0001), current smoker (aHR, 1.187; 95% CI, 1.127-1.250; *P* = 0.0013) or former smoker (aHR, 1.127; 95% CI, 1.048-1.211; *P* < 0.0001), and subjects living in capital city (aHR, 1.037; 95% CI, 1.002-1.073; *P* = 0.0378) were potential confounders for cancer development (**Supplementary Table 1**).

**Table:** Table 1 Baseline characteristics of the study population (n = 713,201).

Characteristics	Control (n = 660,126)	Periodontitis (n = 53,075)	*P* value
**Age, years**	31 (15-46)	49 (39-60)	<0.0001
**CCI score**	0 (0-1)	1 (0-2)	<0.0001
**Sex (%)**			<0.0001
Male	325,796 (49.6)	27,310 (51.5)	
Female	334,330 (50.4)	25,765 (48.5)	
**BMI, kg/m^2^**			<0.0001
< 20	89,951 (13.6)	2,172 (4.1)	
20 ~ 25	80,501 (12.2)	13,088 (24.7)	
≥ 25	41,969 (6.4)	7,534 (14.2)	
Missing	519,496 (78.7)	30,281 (57.1)	
**Smoking**			<0.0001
None	89,951 (13.6)	14,300 (26.9)	
Former	12,049 (1.8)	2,444 (4.6)	
Current	36,498 (5.5)	5,732 (10.8)	
Missing	521,628 (79.0)	30,599 (57.7)	
**Level of income**			<0.0001
≤ 50% (lower income)	256,969 (39.5)	18,910 (35.8)	
51~80%	226,055 (34.8)	17,244 (32.6)	
≥ 81% (higher income)	166,857 (25.7)	16,748 (31.7)	
**Residential area**			<0.0001
Capital city	133,337 (20.2)	11,610 (21.9)	
Urban area	171,117 (25.9)	14,350 (27.0)	
Rural area	255,672 (53.9)	27,115 (51.1)	

### Incidences of overall cancer incidence in patients with periodontitis

**Figure 2:**
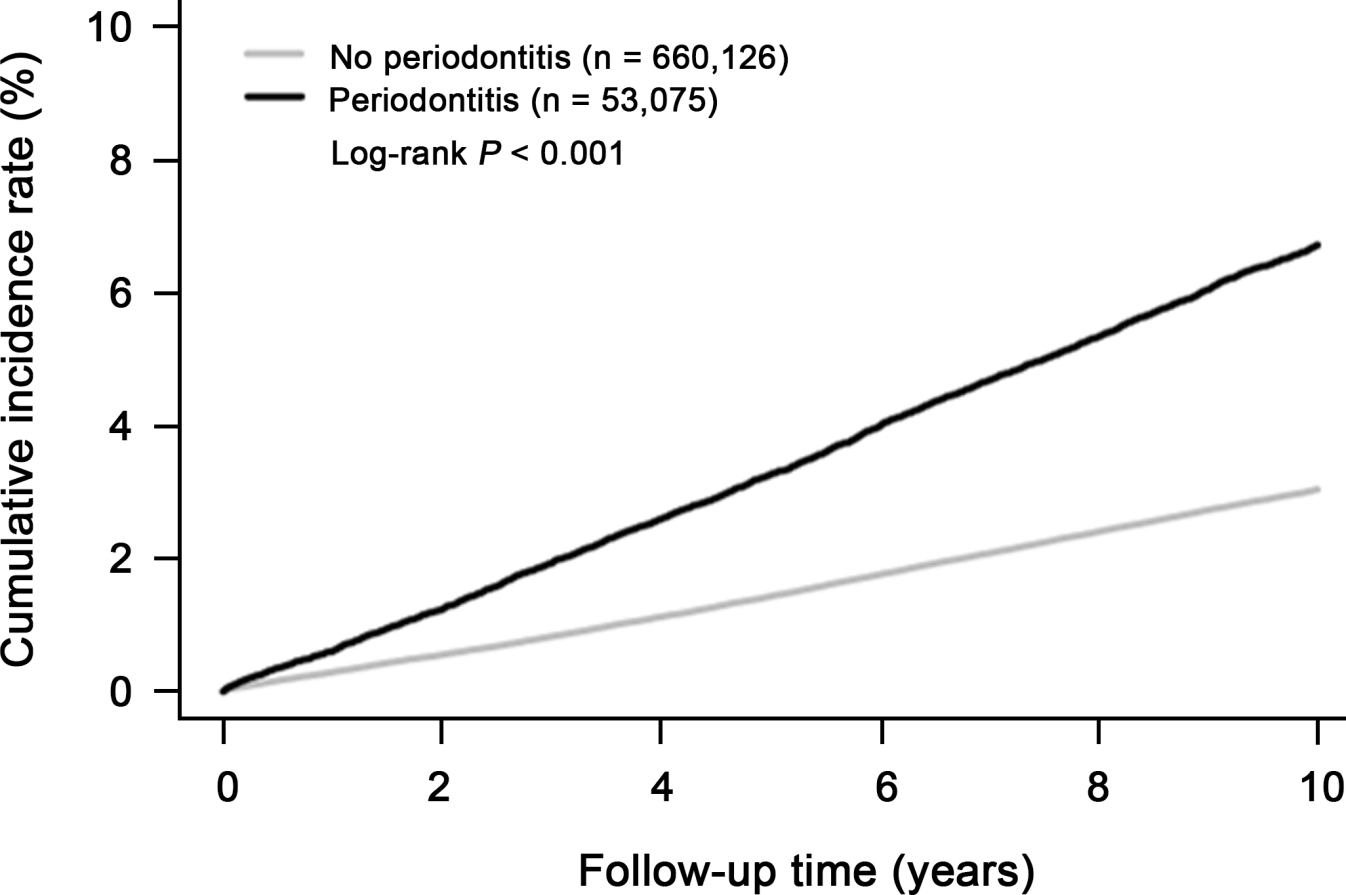
Cumulative incidence of overall cancer in subjects with periodontitis (n=53,075) and without periodontitis (n=660,126).

Next, we evaluated whether periodontitis is associated with increased overall cancer incidence over time. First, we calculated cumulative cancer incidence over time in the subjects with periodontitis, compared to the control group for ten years. One minus the Kaplan-Meier estimate provided an estimate of the cumulative cancer incidence over time in **Figure 2**. Interestingly, the cumulative incidence of cancer in the periodontitis group was 2.2 times higher than that in the control group over ten years (**Figure 2**; log-rank test, *P* < 0.001). The increased incidence rate showed a linear trend over time in periodontitis group (**Figure 2**).

### Risks of organ-specific cancers in patients with periodontitis

To compare the risk of cancer development in the periodontitis and control groups, multivariable Cox proportional hazards regression analysis was performed (**Table 2**). The model was adjusted for potential confounders, such as age, sex, comorbidities defined by the Charlson Comorbidity Index, BMI, smoking history, the level of income, and residential areas (**Table 2**). The overall cancer risk was significantly higher in the periodontitis group than in the control group (aHR 1.129; 95% CI, 1.089-1.171; *P* < 0.0001). Further, diagnosis with periodontitis was associated with increased risks of stomach cancer (aHR, 1.136; 95% CI, 1.042-1.239; *P* = 0.0037), colon cancer (aHR, 1.129; 95% CI, 1.029-1.239; *P* = 0.0105), lung cancer (aHR, 1.127; 95% CI, 1.008-1.260; *P* = 0.0353), bladder cancer (aHR, 1.307; 95% CI, 1.071-1.595; *P* = 0.0085), thyroid cancer (aHR, 1.191; 95% CI, 1.085-1.308; *P* = 0.0002), and leukemia (aHR, 1.394; 95% CI, 1.039-1.872; *P* = 0.0270). Cumulative cancer incidence over time was shown in primary cancer of the stomach, colon, lung, bladder, thyroid, and leukemia, respectively (**Supplementary Figure 1**), suggesting that patients with periodontitis increased overall cancer incidence compared to the control group.

**Table:** Table 2 Adjusted risks of total and organ-specific cancers in patients with periodontitis after correcting confounding factors.

Type of cancer (ICD code)	Number of events			Adjusted HR(95% CI)	*P* value
	Total	Control	Periodontitis		
**Lip, oral cavity, pharynx (C00-C14)**	372	324	48	0.914 (0.670-1.249)	0.5730
**Esophagus (C15)**	314	262	52	1.098 (0.812-1.486)	0.5441
**Stomach (C16)**	3,920	3,278	642	1.136 (1.042-1.239)	0.0037**
**Colon (C18-C20)**	3,416	2,868	548	1.129 (1.029-1.239)	0.0105*
**Liver (C22)**	2,002	1,673	329	1.108 (0.982-1.250)	0.0962
**Gallbladder, biliary tract (C23-C24)**	565	469	96	1.171 (0.937-1.464)	0.1655
**Pancreas (C25)**	674	572	102	1.012 (0.815-1.256)	0.9152
**Larynx (C32)**	157	130	27	1.024 (0.673-1.559)	0.9102
**Lung (C33-C34)**	2,259	1,874	385	1.127 (1.008-1.260)	0.0353*
**Kidney (C64)**	490	404	86	1.249 (0.984-1.584)	0.0676
**Bladder (C67)**	602	475	127	1.307 (1.071-1.595)	0.0085**
**Brain, CNS (C70-C72)**	402	353	49	1.152 (0.848-1.566)	0.3662
**Thyroid (C73)**	4,399	3,871	528	1.191 (1.085-1.308)	0.0002**
**Hodgkin lymphoma (C81)**	37	34	3	0.956 (0.283-3.229)	0.9425
**Non-Hodgkin lymphoma (C82-C86, C96)**	497	430	67	1.045 (0.803-1.359)	0.7439
**Multiple myeloma (C90)**	137	114	23	1.189 (0.754-1.874)	0.4567
**Leukemia (C91-C95)**	399	344	55	1.394 (1.039-1.872)	0.0270*
**Other malignant neoplasms (remainder of C00-C96)**	5,322	4,565	757	1.102 (1.019-1.192)	0.0148*
**Total**	**23,281**	**19,778**	**3,503**	**1.129 (1.089-1.171)**	<0.0001**

*P < 0.05; **P < 0.01.

Next, we evaluated aged-matched adjusted hazard ratio in subjects with ≥40 years old from the study population (**Supplementary Table 2**). An increased risk of total cancer (aHR 1.080; 95% CI, 1.040-1.122; *P* < 0.0001) was observed for the periodontitis group compared with the control group (**Supplementary Table 2**). By cancer site, significant associations for periodontitis groups were observed for bladder cancer (aHR, 1.307; 95% CI, 1.069-1.598; *P* = 0.0091), thyroid cancer (aHR, 1.123; 95% CI, 1.008-1.251; *P* = 0.0349), and leukemia (aHR, 1.407; 95% CI, 1.016-1.947; *P* = 0.0396), respectively.

### Risks of secondary malignancy in patients with periodontitis

Finally, we evaluated whether periodontitis affects the occurrence of secondary cancer in cancer survivors. The incidence rates of secondary cancer were 0.89% (176 out of 19,778) and 1.03% (36 out of 3,503) in the control and periodontitis groups, respectively (**Supplementary Table 3**). Although the incidence rates of secondary malignancy are 0.14% higher in the periodontitis group, the difference was not statistically significant (*P* = 0.428). Collectively, there was no significant association between the development of secondary malignancy and the history of periodontitis in cancer survivors who were alive five years after they were diagnosed with the primary malignancy.

## Discussion

In this study, we showed that patients with periodontal disease, including periodontitis, have an increased overall cancer incidence and an organ-specific cancer incidence compared to control individuals. Periodontitis was associated with increased risks of gastrointestinal cancers (such as stomach cancer, colon cancer), lung cancer, bladder cancer, thyroid cancer and leukemia. Even after controlling for confounding factors, such as sex, income, smoking history, BMI, and comorbidities, periodontitis was found to be a modest but obvious risk factor for cancer.

Previous observational reports and meta-analyses suggested that periodontal disease was associated with increased risks of several cancer types, including head and neck, lung, pancreatic, colorectal, kidney, and hematologic cancers  (8, 12, 13). Our results are consistent and comparable with published data. For instance, previous studies showed periodontitis was positively correlated with an increased risk of lung cancer (HR, 2.33; 95% CI, 1.51 to 3.60) and colon cancer among never smokers (HR, 2.12; 95% CI, 1.00 to 4.47) (13). Our finding is consistent with the previous reports on colon cancer (adjusted HR, 1.129; *P*=0.011) and lung cancer (adjusted HR, 1.127; *P*=0.035). Although we did not observe a positive correlation between periodontitis and increased risk of head and neck cancers (adjusted HR, 0.914; *P*=0.573), the number of head and neck cases was small to draw a conclusion in this study. Future larger association study is warranted. Interestingly, our study showed a strong correlation with the development of inflammation-associated cancers, such as bladder cancer (adjusted HR, 1.307; *P*=0.008) and thyroid cancer (adjusted HR, 1.191; *P*<0.001) after correction of smoking history.

The potential relationship between periodontitis and cancer can be explained by the properties of local and systemic inflammation associated with bacteremia and increased myelopoietic activity  (7). Periodontitis causes increased systemic inflammation because of increased bacterial infection, hematogenous dissemination of oral pathogenic bacteria, increased inflammatory mediators (such as interleukin [IL]-1, IL-6, and C-reactive protein and fibrinogen), and increased neutrophil number in the bloodstream  (19–21). Chronic systemic inflammation causes cellular stress, including DNA damage through reactive oxygen species stress and reactive nitrogen species  (22). Further, inflammatory mediators like NF-κB and STAT3 increase genetic instability. Additionally, repeated tissue damage and repair trigger chromosomal translocation. These mechanisms induce DNA damage and mutation. Inflammation and genetic instability have a sufficiently significant causal association for inflammation to be included as a hallmark of cancer  (23, 24).

Another explanation for the relationship between periodontitis and cancer is oral bacteria  (21). Frequent transient bacteremia of oral pathogens leading to sustained systemic inflammatory responses appears to be key to the mechanism of carcinogenesis in patients with chronic periodontitis  (25). Periodontitis can also cause oral and gut dysbacteriosis. *Porphyronas gingivalis* (*P. gingivalis*) infection can alter the gut microbiota, enhance blood endotoxin levels, cause systemic inflammation, interfere with the host metabolism, and promote immune system evasion  (21, 26–28). *P. gingivalis* has been shown to evade innate immune detection and enhance chronic inflammation of vascular structures through TLR-4  (21, 28). Patients with oral diseases such as gingivitis and periodontitis may be more likely to develop intestinal dysbiosis  (29, 30). *P. gingivalis* is also found in patients with colorectal cancer, and human colon cells infected by *P. gingivalis* can develop into colorectal cancer  (30). Moreover, oral bacteria such as *Gemella, Peptostreptococcus*, and *Fusobacterium* are strongly correlated with colorectal cancer  (31). Many studies have proposed the association between colon cancer development by *Fusobacterium nucleatum (F. nucleatum)*  (32–34). *F. nucleatum* binds to tumor cells *via* the virulent adhesin protein Fap2 and activates Wnt signaling pathway, leading to epithelial-mesenchymal transition  (35). Furthermore, direct interaction between the FadA adhesin proteins and E-cadherin on the surface of colonic epithelial cells increased E-cadherin/β-catenin-modulated transcription factors, leading to DNA damage, epithelial cell proliferation, and acquisition of cancer stemness.

This study has several limitations. First, previous studies suggest a positive correlation between periodontitis and breast and genitourinary cancers  (36–39). Unfortunately, the National Health Insurance Database does not classify reproductive organ carcinomas such as breast and genitourinary cancers but categorizes them as “other” cancers. Although we could not evaluate the association between periodontitis and breast cancer and genitourinary incidence, we included them in the overall cancer incidence calculation. Second, because the NHIS database does not classify the severity of periodontitis (such as the number of teeth affected), we could not evaluate the association of periodontitis severity or treatment history with cancer risk  (13). Third, the lack of circulating markers or bacterial levels in the NHIS database does not allow further analysis to identify the role of specific oral microbiota in cancer development. Forth, the heterogeneity nature of the ICD-10 diagnosis code for periodontitis and cancer diagnosis may lead to selection bias and underestimate the association in this study. Of note, this study based on ICD-10 codes does not reflect recently updated periodontitis classification criteria  (3). A prospective cohort using the updated periodontal disease classification criteria study will increase the accuracy of the analysis. Fifth, a large portion of missing information in smoking history and body mass index is a potential bias for adjusting for confounding factors. We collected smoking history and body mass index [BMI] based on the patient-reported healthy examination questionnaire in the patient cohort. However, many subjects in the patient cohort had missing information on the healthy examination questionnaire. In addition, although we confirmed the primary tumor site, the pathological findings were not available from the National Health Insurance Database. Therefore, it was not possible to assess the association between the pathological characteristics of periodontitis and cancer.

In conclusion, periodontal disease, including periodontitis, was associated with increased risk of cancer, which persisted after controlling for confounding factors. Further prospective research is warranted to establish a causal relationship.

## Data availability statement

Data access to the NHIS database enables via National Health Insurance Sharing Service (https://nhiss.nhis.or.kr/bd/ay/bdaya001iv.do). The application form, a research proposal, and an approval document from the appropriate IRB be submitted to and reviewed by the NHIS inquiry committee for research support.

## Ethics statement

The studies involving human participants were reviewed and approved by Severance Hospital Human Research Protection Center (Institutional Review Board No. 4-2019-0616). Written informed consent from the participants’ legal guardian/next of kin was not required to participate in this study in accordance with the national legislation and the institutional requirements.

## Author contributions

HK, IJ, EK, and ML contributed to the conception, design, and the interpretation of the study. EHK collected and analyzed the data. HK, SN, EK, and CP reviewed, interpreted, and drafted the manuscript. YK, JA, SS, YP, HJ, and B-IK reviewed and edited the manuscript. All the authors read and approved the final manuscript for submission and take responsibility for the data presented in this manuscript.

## Funding

This study was supported in part by the National Research Foundation of Korea (NRF) grant funded by the Korea government (MSIT) (No. 2022R1A2C4001879 to H.S.K.), the Bio & Medical Technology Development Program of the National Research Foundation (NRF) & funded by the Korean government (MSIT) (No. 2022M3A9F3016364 to H.S.K.), the Korea Health Technology R&D Project through the Korea Health Industry Development Institute (KHIDI), funded by the Ministry of Health & Welfare, Republic of Korea (No. HI21C0974 to H.S.K.), and a faculty research grant of Yonsei University College of Medicine (6-2020-0218 to H.S.K.).

## Acknowledgments

The content of this manuscript has been presented in part at the European Society for Medical Oncology (ESMO) in 2019.

## Conflict of interest

The authors declare that the research was conducted in the absence of any commercial or financial relationships that could be construed as a potential conflict of interest.

## Publisher’s note

All claims expressed in this article are solely those of the authors and do not necessarily represent those of their affiliated organizations, or those of the publisher, the editors and the reviewers. Any product that may be evaluated in this article, or claim that may be made by its manufacturer, is not guaranteed or endorsed by the publisher.

## Supplementary material

Supplementary Figure 1 Cumulative incidence in primary cancer of the stomach, colon, lung, bladder, thyroid, and leukemia in subjects with periodontitis (n=53,075) and without periodontitis (n=660,126).
