# Supplementary figures and images for: Periodontal disease and cancer risk: A nationwide population-based cohort study
Source: Front Oncol. 2022 Aug 23;12:901098. doi: 10.3389/fonc.2022.901098 (PMC9445882; doi:10.3389/fonc.2022.901098)

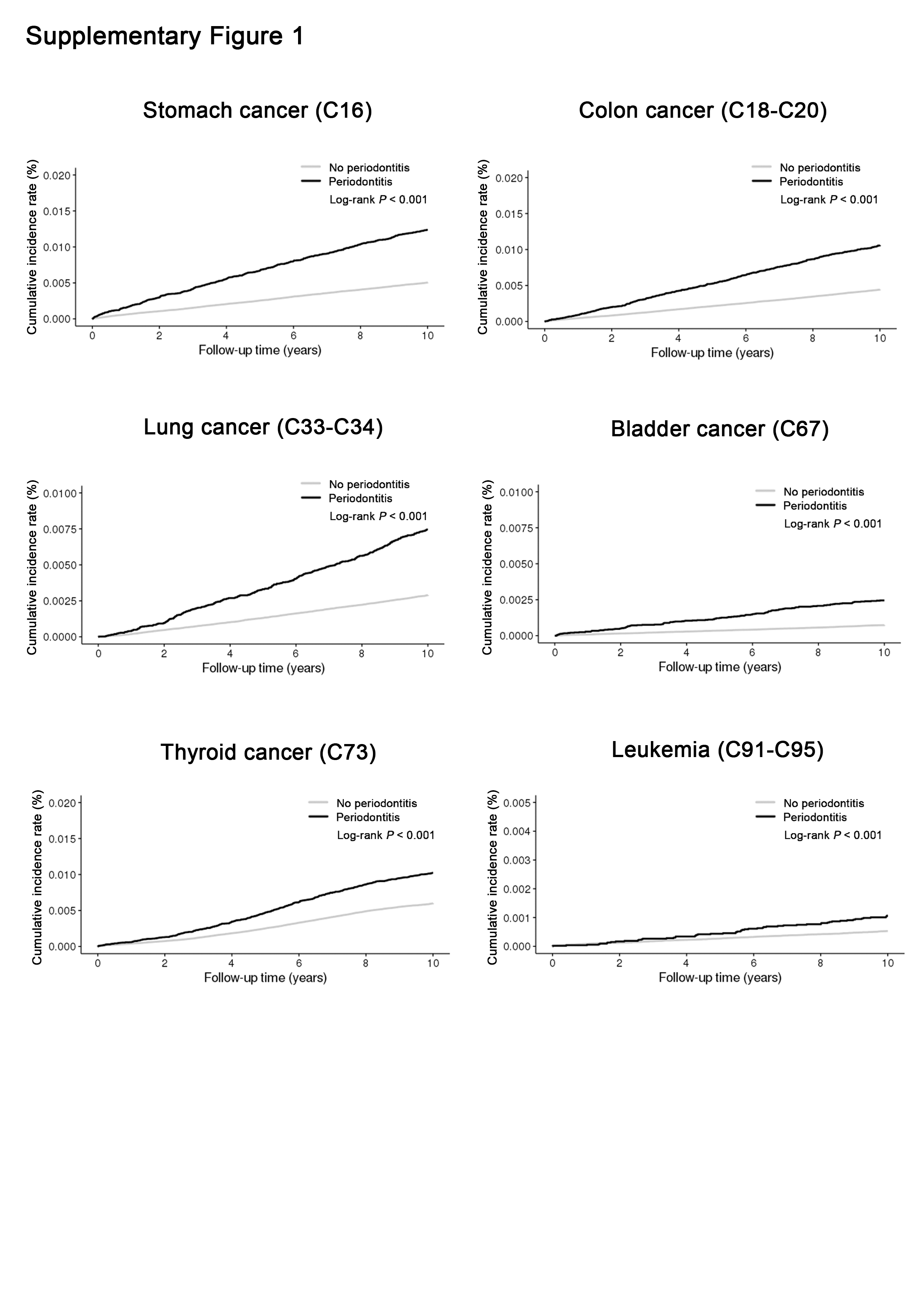

Supplement: Supplementary Figure 1 — Cumulative incidence in primary cancer of the stomach, colon, lung, bladder, thyroid, and leukemia in subjects with periodontitis (n=53,075) and without periodontitis (n=660,126). [file Image_1.tif]
