# Supplementary material for: Periodontal disease and cancer risk: A nationwide population-based cohort study
Source: Front Oncol. 2022 Aug 23;12:901098. doi: 10.3389/fonc.2022.901098 (PMC9445882; doi:10.3389/fonc.2022.901098)
Supplement: Supplementary file 2 [file Table_1.docx]

Supplementary Material

**Supplementary Table 1. Multivariate Cox regression analysis of potential confounding factors for cancer development in this study cohort (n = 713,201).**

| **Characteristics** | **Adjusted HR (95% CI)** | ***P* value** |
| --- | --- | --- |
| **Age, years** | 1.058 (1.057-1.059) | < 0.0001 |
| **CCI score** | 1.054 (1.044-1.063) | < 0.0001 |
| **Sex (%)** |  |  |
| Male | 1 (reference) |  |
| Female | 0.761 (0.740-0.783) | < 0.0001 |
| **BMI, kg/m^2^** |  |  |
| < 20 | 1.032 (0.962-1.108) | 0.3772 |
| 20 ~ 25 | 1 (reference) |  |
| ≥ 25 | 0.974 (0.930-1.019) | 0.2508 |
| Missing | 0.946 (0.806-1.112) | 0.5025 |
| **Smoking** |  |  |
| None | 1 (reference) |  |
| Former | 1.127 (1.048-1.211) | 0.0013 |
| Current | 1.187 (1.127-1.250) | < 0.0001 |
| Missing | 0.998 (0.850-1.173) | 0.9824 |
| **Level of income** |  |  |
| ≤ 50% (lower income) | 0.993 (0.962-1.024) | 0.6385 |
| 51~80% | 1 (reference) |  |
| ≥ 81% (higher income) | 0.994 (0.961-1.028) | 0.7353 |
| **Residential area** |  |  |
| Capital city | 1.037 (1.002-1.073) | 0.0378 |
| Urban area | 1.022 (0.990-1.055) | 0.1750 |
| Rural area | 1 (reference) |  |
