# Supplementary material for: Periodontal disease and cancer risk: A nationwide population-based cohort study
Source: Front Oncol. 2022 Aug 23;12:901098. doi: 10.3389/fonc.2022.901098 (PMC9445882; doi:10.3389/fonc.2022.901098)
Supplement: Supplementary file 3 [file Table_2.docx]

Supplementary Material

**Supplementary Table 2. Adjusted risks of total and organ-specific cancers in patients with periodontitis after correcting confounding factors in subjects with ≥40 years old.**

| **Type of cancer (ICD code)** | **Number of events** | | | **Adjusted HR**  **(95% CI)** | ***P* value** |
| --- | --- | --- | --- | --- | --- |
|  | **Total** | **Control** | **Periodontitis** |  |  |
| **Lip, oral cavity, pharynx (C00-C14)** | 321 | 277 | 44 | 0.839 (0.605-1.162) | 0.2897 |
| **Esophagus (C15)** | 306 | 254 | 52 | 1.067 (0.788-1.445) | 0.6753 |
| **Stomach (C16)** | 3,611 | 2,987 | 624 | 1.073 (0.983-1.171) | 0.1173 |
| **Colon (C18-C20)** | 3,148 | 2,618 | 530 | 1.068 (0.971-1.174) | 0.1738 |
| **Liver (C22)** | 1,843 | 1,522 | 321 | 1.076 (0.952-1.216) | 0.2422 |
| **Gallbladder, biliary tract (C23-C24)** | 552 | 457 | 95 | 1.147 (0.916-1.436) | 0.2321 |
| **Pancreas (C25)** | 641 | 541 | 100 | 0.988 (0.794-1.229) | 0.9146 |
| **Larynx (C32)** | 156 | 129 | 27 | 0.983 (0.646-1.496) | 0.9374 |
| **Lung (C33-C34)** | 2,199 | 1,818 | 381 | 1.100 (0.983-1.231) | 0.0962 |
| **Kidney (C64)** | 410 | 328 | 82 | 1.207 (0.944-1.543) | 0.1333 |
| **Bladder (C67)** | 573 | 447 | 126 | 1.307 (1.069-1.598) | 0.0091** |
| **Brain, CNS (C70-C72)** | 250 | 207 | 43 | 1.234 (0.885-1.722) | 0.2153 |
| **Thyroid (C73)** | 2,714 | 2,315 | 399 | 1.123 (1.008-1.251) | 0.0349* |
| **Hodgkin lymphoma (C81)** | 14 | 11 | 3 | 1.584 (0.434-5.778) | 0.4857 |
| **Non-Hodgkin lymphoma (C82-C86, C96)** | 369 | 308 | 61 | 1.038 (0.786-1.371) | 0.7901 |
| **Multiple myeloma (C90)** | 132 | 110 | 22 | 1.119 (0.703-1.780) | 0.6360 |
| **Leukemia (C91-C95)** | 233 | 186 | 47 | 1.407 (1.016-1.947) | 0.0396* |
| **Other malignant neoplasms (remainder of C00-C96)** | 4,560 | 3,831 | 729 | 1.106 (1.021-1.199) | 0.0138* |
| **Total** | **19,589** | **16,315** | **3,274** | **1.080 (1.040-1.122)** | **<0.0001**** |

# **P* < 0.05; ***P* < 0.01
