# Supplementary material for: Periodontal disease and cancer risk: A nationwide population-based cohort study
Source: Front Oncol. 2022 Aug 23;12:901098. doi: 10.3389/fonc.2022.901098 (PMC9445882; doi:10.3389/fonc.2022.901098)
Supplement: Supplementary file 4 [file Table_3.docx]

Supplementary Material

**Supplementary Table 3. The incidence of secondary cancer 5 years after diagnosis of primary cancer**

|  | **Control** | **Periodontitis** |
| --- | --- | --- |
| **Total** | 176 | 36 |
| **Not recurrence** | 154 | 29 |
| **Recurrence** | 22 | 7 |
| Colon(C18-C20) | 2 | 1 |
| Gallbladder, biliary tract (C23-C24) | 1 | 0 |
| Brain, CNS(C70-C72) | 1 | 0 |
| Leukemia(C91-C95) | 2 | 0 |
| Others (Re. C00-C96) | 16 | 6 |

*P* = 0.4287
